# How and When? Metacognition and Solution Timing Characterize an “Aha” Experience of Object Recognition in Hidden Figures

**DOI:** 10.3389/fpsyg.2019.01023

**Published:** 2019-05-22

**Authors:** Tetsuo Ishikawa, Mayumi Toshima, Ken Mogi

**Affiliations:** ^1^Medical Sciences Innovation Hub Program, RIKEN, Yokohama, Japan; ^2^Digital Content and Media Sciences Research Division, National Institute of Informatics, Tokyo, Japan; ^3^Sony Computer Science Laboratories, Inc., Tokyo, Japan

**Keywords:** “aha!” experience, insight problem solving, suddenness, pleasure, confidence, recognition time, metacognition, hidden figure

## Abstract

The metacognitive feelings of an “aha!” experience are key to comprehending human subjective experience. However, behavioral characteristics of this introspective cognition are not well known. An aha experience sometimes occurs when one gains a solution abruptly in problem solving, a subjective experience that subserves the conscious perception of an insight. We experimentally induced an aha experience in a hidden object recognition task, and analyzed whether this aha experience was associated with metacognitive judgments and behavioral features. We used an adaptation of Mooney images, i.e., morphing between a grayscale image and its binarised image in 100 steps, to investigate the phenomenology associated with insight: aha experience, confidence, suddenness, and pleasure. Here we show that insight solutions are more accurate than non-insight solutions. As metacognitive judgments, participants’ confidence in the correctness of their solution is higher in insight than non-insight problem solving. Intensities of the aha feeling are positively correlated with subjective rating scores of both suddenness and pleasure, features that show marked signs of unexpected positive emotions. The strength of the aha experience is also positively correlated with response times from the onset of presentation until finding the solution, or with task difficulty only if the solution confidence is high enough. Our findings provide metacognitive and temporal conditions for an aha experience, characterizing features distinct from those supporting non-aha experience.

## Introduction

Cognitive findings are sometimes accompanied by particular experiences, just as in ancient Greek Archimedes exclaimed “eureka!” to express his delight of a scientific discovery. This phenomenon is called “aha!” experience (Gick and Lockhart, 1995; Topolinski and Reber, 2010; Webb et al., 2018). In the context of problem solving and creative thinking, “aha!” or “eureka!” experience is thought to be a synonym of insight (Weisberg, 2015), defined as a sudden change in knowledge representation or the rapid formation of a new concept, often leading to the solution of a problem (Kounios and Beeman, 2014). This insight frequently elicits a burst of various emotions (Shen et al., 2016), including a positive surprise at either the content or the way of realization. Solution accuracies with aha experience tend to be higher than those without aha (Salvi et al., 2016; Webb et al., 2016; Danek and Wiley, 2017). Unexpected transition from disfluency to fluency, or abrupt switch from incorrect solution into correct solution tends to induce stronger aha experience. Social psychologist Robert Cialdini described the tendency as follows: “the *Aha!* experience is much more satisfying when it is preceded by the *Huh?* experience” (Heath and Heath, 2007; Webb et al., 2019).

Major theories of aha experience have proposed two key aspects: (i) appropriate (desirable) difficulty (Hebb, 1949) and (ii) cognitive fluency (Topolinski and Reber, 2010; in more general context, see also, Oppenheimer, 2008). According to the appropriate difficulty theory, insight tends to occur when the problem is neither too easy nor too difficult. In other words, if the task is too difficult it is impossible to solve the problem, and if it is too easy there is no surprise. According to the fluency theory, even if the problem is difficult, if cognitive processes at the moment of solution is fluent, the solution with high confidence often tends to be correct, accompanied by positive emotions, or surprise “aha!”. In line with the fluency theory, the assumption that confidence in insight solutions is greater than that of non-insight ones is sometimes called the confidence hypothesis (Danek et al., 2014b). In the same vein, the supposition that accuracy of insight solutions is higher than that of non-insight ones is referred to as the accuracy hypothesis (Salvi et al., 2016; Danek and Salvi, 2018).

The accuracy advantage of the aha is related to metacognition, i.e., meta-level processes of “cognition about cognition” or “knowing about knowing.” Metacognitive feelings leading to the right answers with the aha could be characterized by two stages: metacognition before reaching the solution and metacognition after the eureka moment. In the field of insight problem solving, metacognitive sense about the psychological distance from a solution is often assessed by warmth rating, or “Feeling of Warmth” (FoW), applying thermal metaphor to express feelings of near and distant as “hot” and “cold,” respectively (Metcalfe, 1986). When subjects have a certain metacognitive sense that “the solution is near” (i.e., high FoW) long before a solution moment or metacognitive feelings are gradually changing (i.e., gradual increase in the FoW) as to approach a solution, the answer is likely to be a false alarm, or wrong answer (Metcalfe, 1986), the solution accuracy tending to be low. In such cases, the solving process may be judged as a non-insight. On the other hand, when subjects have a characteristic metacognitive feeling (i.e., abrupt jump of the FoW from cold to hot) just at the moment of realization of an answer, the solution process may be judged as an insight (Metcalfe and Wiebe, 1987; Kizilirmak et al., 2018; but see also, Hedne et al., 2016; Laukkonen and Tangen, 2018). If metacognitive feelings of confidence about the solution rapidly increase at the eureka moment, and neither additional deliberate reasoning, analytical validation, nor feedback of answer correctness would not be needed, it would be suggested that the confidence hypothesis and the accuracy hypothesis are both true.

Cognitive processes accompanied by aha experiences typically result in stronger long-term memory (“memory advantage”) than cases of solution without aha (Danek et al., 2013; Kizilirmak et al., 2016). After an insightful realization, the learned knowledge sometimes prevents subjects from going back to the previous naïve state. This unique type of learning is a long-term memory encoding of one-shot experience (Ludmer et al., 2011), or called “one-shot learning” (Giovannelli et al., 2010; Ishikawa and Mogi, 2011; Dudai and Morris, 2013).

A typical example of such irreversible cognitive processes of one-shot learning is the visual object recognition in hidden figures. The hidden figure consisting of a grayscale or black-and-white high contrast ambiguous picture such as “Cow” (Dallenbach, 1951) and “Dalmatian” (Gregory, 1970) seems to be meaningless blobs (Ishizu, 2013) or meaningful but nonholistic pareidolias (Taylor et al., 2017) for naïve viewers. Once the viewers realize what is concealed in the hidden figure with appropriate disambiguation (e.g., interpretation in a sensible way; Hegdé and Kersten, 2010; Ishizu, 2013), sometimes an insightful “aha!” moment comes with a pleasant sensation.

If one suddenly reaches a plausible interpretation of the hidden figure on one’s own competence, generating a solution with aha, one would typically have more positive emotions accompanied by stronger memory about the solution than when aha occurs without generating the answer (Kizilirmak et al., 2016). The solutions with aha would be remembered better than those without aha (Danek et al., 2013).

Generating a solution in hidden figure perception is characterized by the process of spontaneously and consciously becoming aware of the answer. To fully investigate the phenomenology of conscious awareness in hidden figures, simultaneous measurement of both objective and subjective indices are needed to characterize the phenomena. However, most of the previous studies investigating the cognitive mechanisms of object detection and recognition in hidden figures did not explicitly measure any subjective indices of the phenomenological aspects of aha experience. Interrelationship between subjective aha feelings and its strength in hidden figure recognition are not well known. Successful cognitive strategies in generating answers and induce desirable aha experience are not yet clear.

A review of major experimental paradigms in previous studies would clarify several limitations of previous experiments with hidden figures as stimuli. The simplest method is to continue presenting a hidden figure as a still image until recognition (Imamoglu et al., 2012; Murata et al., 2014). In this “no change paradigm (NCP),” the task difficulty is not easy to adjust properly, as the process of utilizing the combination of blurring and thresholding to create a hidden figure often makes the image too hard or too easy to recognize. If the problem is too difficult to solve, the answer rate within a certain period of time decreases, while the responded data available to analyze also decreases (Ishikawa and Mogi, 2011). On the other hand, if the problem is too easy, there is no stagnation (“impasse”) in the first place, compromising the suitability as a problem-solving task (Salvi et al., 2016). Secondly, there is a more sophisticated method in which the stimulus image suddenly switches from a two-tone (hidden figure) to a grayscale (“answer”) photograph after being presented for a certain predetermined duration (Dolan et al., 1997; Ludmer et al., 2011; Kizilirmak et al., 2016). In this “rapid change paradigm (RCP),” where the answer is directly exposed all of a sudden, the viewer can be forcedly made to recognize the answer. Although the RCP guarantees higher answer rate than in the case of the NCP, it eliminates or at least alleviates the cognitive processes of solving spontaneously without seeing the answer. Furthermore, the RCP would not facilitate investigations into conditions of the spontaneous occurrence and timing of aha. In sum, neither the NCP nor RCP provides a sufficiently robust method for studying aha experience in an experimentally tangible manner.

In order to solve these problems, an experimental methodology (Ishikawa and Mogi, 2011) was developed by morphing from black and white binarized images to grayscale images by generating images at intermediate stages, arranging them in order in frames and making them animated from the problem (hidden figure) toward the answer (grayscale “original”), thus producing a gradually changing stimulus (Gradual change paradigm, or GCP).

Firstly, to validate that insight would be induced by the GCP in hidden figures, we would confirm two auxiliary hypotheses from theories of insight,: (i) accuracy hypotheses (Salvi et al., 2016; Danek and Salvi, 2018) predicting that insight solutions are more accurate than non-insight solutions and (ii) confidence hypothesis (Danek et al., 2014b) predicting that “Participants’ confidence in the correctness of their solution differs between insight and noninsight problem solving.” After the validity checks, by combining appropriate (desirable) difficulty theory (Hebb, 1949) and cognitive fluency theory (Topolinski and Reber, 2010), we tested a main hypothesis that if the hidden figure problem is not too easy, and the final confidence is high enough, the high fluency at the insightful moment would induce a strong sense of aha accompanied by positive emotions. We confirm these hypotheses, shedding light on the conditions under which an aha experience would occur.

## Materials and Methods

### Participants

Ten naïve adults (six females and four males, mean ± SD age: 33 ± 6 years old) participated in the experiment. All participants had a normal or corrected-to-normal visual acuity. This study was carried out in accordance with the recommendations of The Brain and Cognitive Sciences Ethics Committee of Sony Computer Science Laboratories with written informed consent from all subjects. All subjects gave written informed consent in accordance with the Declaration of Helsinki. The protocol was approved by The Brain and Cognitive Sciences Ethics Committee of Sony Computer Science Laboratories.

### Procedure

**FIGURE 1:**
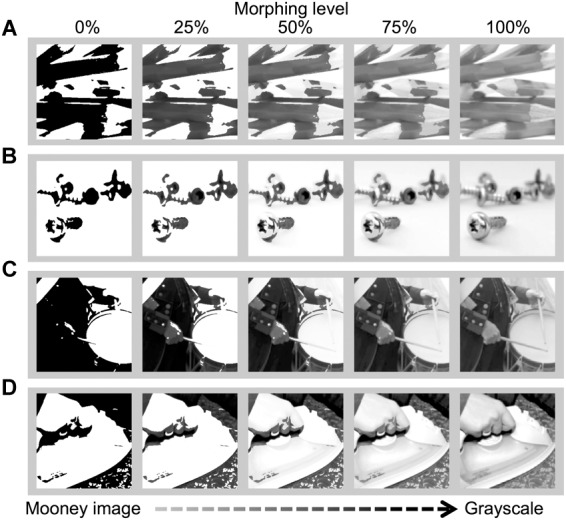
Movie frames of representative stimuli inducing strong aha **(A,B)** and weak aha feelings **(C,D)**. **(A)** The highest aha score (mean = 4.9) image with high confidence score (= 5.5) and long RT (median ML = 98.2%). **(B)** Moderately high aha score (= 4.0) image with high confidence (= 5.6) and long RT (ML = 87.4%). **(C)** Fairly-low aha score (= 2.8) image with high confidence (= 5.7) and short RT (ML = 10.9%). **(D)** Considerably low aha score (= 2.7) image with low confidence (= 4.2) and relatively long RT (ML = 77.4%). Note that the morphing movie frames change from the left to right. Correct answers: **(A)** Pencils, **(B)** screws, **(C)** a drum, and **(D)** an iron. The original objects before degradation were adapted from http://www.freeimages.com/.

Sixty-five movie stimuli were created by means of the morphing paradigm (Ishikawa and Mogi, 2011). Each movie was constructed as follows. An 8-bit (0–255 levels) grayscale picture of common object(s) was cropped to 300 pixels × 300 pixels size, and blurred (Gaussian filter: radius = 3 pixels) and binarized (128 for the threshold value). The resultant figure was a black and white two-tone image, or a “Mooney image” (Mooney and Ferguson, 1951; Mooney, 1957). A morphing technique using the software Norrkross MorphX (Norrkross Software, Tynningö, Sweden), was applied to the Mooney image and its original blurred counterpart. Morphing levels (MLs) defined by the percentage of the blurred grayscale image in the fusion image was an index of degradation. Finally, one hundred and one degraded images (of MLs from 0 to 100%, with increments of 1%) were converted into a movie with 101 frames in total. In the movie, frames were presented in the ascending direction from 0 (Mooney image) to 100% (blurred grayscale image). Example movie frames extracted from four representative stimuli are shown in Figure 1. All objects were selected from the normative set (Snodgrass and Vanderwart, 1980). The frame rate of the movie was 10 fps (100 ms/frame) or 5 fps (200 ms/frame). The relevant measure of movie replay speed was percent ML increment per second (%/s). The change speed of stimuli was 10 %/s or 5 %/s, with a total duration of 10.1 and 20.2 s, respectively.

### Stimuli

**FIGURE 2:**
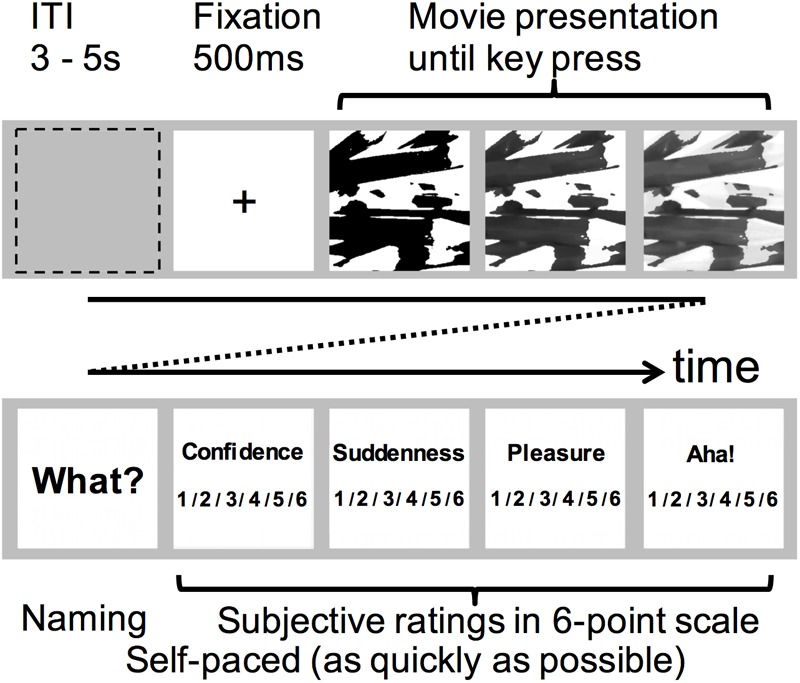
Experimental procedure denoting a single trial time course, consisting of two stages: The stimulus presentation phase (upper row) and the answering phase (bottom row). In the first stage, the participants were required to find hidden object(s) in the movie stimulus. When a “eureka” moment comes, they were instructed to indicate by the [SPACE] key press. In the second stage, after the “What was it?” message was displayed, the participants were asked to verbally report the object name and press the [SPACE] key to proceed. The confidence, suddenness, delight, and “aha!” ratings were reported in a six-point scale by selecting and pressing one of the [1]–[6] keys.

The stimuli were presented on a 13-inch MacBook (Apple Inc., Cupertino, CA, United States) display against middle gray (128 level) background using MATLAB R2010a (The MathWorks, Inc., Natick, MA, United States) with Psychtoolbox (Brainard, 1997; Pelli, 1997). The participants were seated at a distance of 60 cm from the display. The trial timeline was as follows (Figure 2). The fixation cross (“+” mark) was shown for 500 ms at the start of each trial. After the fixation cue disappeared, the morphing movie (visual angle: 10° × 10°) presentation begun. The participants were instructed to press the [SPACE] key as quickly as possible when they recognized object(s) in the movie. We defined response or recognition times (RTs) as the times from the onset of movie presentation to the key press. When the participant pressed the [SPACE] key, the movie disappeared and the display flipped to the next answer step. When the last frame of movie disappeared, the trial continued to the next answer phase. The participants were required to report the object name verbally. Then they were also asked to provide subjective ratings (cf. Danek and Wiley, 2017) in a six-point Likert scale on four types of feelings: (i) Confidence: How sure are you about your solution? (ii) Suddenness: How suddenly did you find the answer?, (iii) Pleasure: How much pleasure did you get?, and (iv) Aha feeling: How strongly did you feel “aha!”? In the six-point Likert scales, the participants were asked to indicate the extent to which they agreed/disagreed with these questionnaires. Greater rating scores corresponded to higher/stronger feelings and smaller rating scores corresponded to lower/weaker feelings. The order of subjective ratings was fixed throughout the experiment to reduce the cognitive demand. The inter-trial interval (ITI) was randomly selected from 3 to 5 s.

In the instruction phase preceding the practice trials, subjects were shown Dallenbach’s Cow and Gregory’s Dalmatian as typical examples of hidden figures inducing aha experiences, and asked to search for hidden objects. If they did not spontaneously recognize answer objects after a while, some hints on the location of objects and (if necessary) object names were provided one after another. If the subjects could not realize how and where objects were hidden after getting some clues, detailed explanation of answers was provided until they were convinced. After that, participants were told that the very experience when the object hidden in such a figure was suddenly and clearly understood was a typical aha experience. Subsequently, in the practice phase, subjects practiced to recognize objects in hidden morphing movies and judge strengths of subjective assessments in six-point Likert scales on (i) aha feeling, (ii) confidence, (iii) suddenness, and (iv) pleasure. If they had any questions or requests for clarification, additional explanations were provided.

Five movies were used for the practice session, while the remaining sixty were reserved for the main experiment. The two alternative replay speeds (10 %/s or 5 %/s) were randomly assigned to half of the trials each and counterbalanced across the participants.

### Statistical Analysis

To compare means for multiple groups, paired *t*-test or type III ANOVA with Kenward–Roger’s method was used. By correcting the degrees of freedom, the latter method can handle missing cases (e.g., data from a participant with no incorrect answers) properly without information loss due to omission. In correlation analysis, Spearman’s rank correlation coefficients (*r*_s_) (which were suitable to estimate monotonic relationships, i.e., robust effect sizes of correlations by reducing possible spurious effects from outliers) and its 95 percent confidence intervals (95% CIs) were evaluated after averaging scores for each stimulus (by-item analysis).

We used the type-2 receiver operating characteristic (ROC) analysis suitable for measuring metacognition (Fleming et al., 2010; Fleming and Lau, 2014). In contrast with the type-1 ROC analysis, the type-2 ROC analysis is similar but meta-level analysis. In other words, the type-2 ROC analysis is an analysis to quantify the precision of metacognition based on the degree of confidence and performance (i.e., accuracy). Fleming et al. (2010) stated that “Participants’ confidence ratings were used to construct a type II ROC function that quantifies the ability to discriminate between correct and incorrect responses cumulated across levels of confidence.” More specifically, the type-2 ROC curves are constructed from false positive and true positive defined by *p*(confidence | incorrect) and *p*(confidence | correct), respectively. Area under the type-2 ROC curve (AUC) was calculated as a measure of metacognitive precision.

In hierarchical regression, we built linear mixed model (LMM) with participants as random effect. In order to correct degrees of freedom in the LMM, we made the Kenward–Roger adjustment. We applied R lmerTest::lmer() function with *post hoc* Tukey’s all-pair multiple comparisons using the multcomp::glht() protocol. The model estimation was optimized in the same way as in Folke et al. (2016). The significance level of any statistical test was set to alpha = 0.05. For correction of multiple comparisons in both the comparisons between groups and the correlation analysis, *p*-values were adjusted by the Holm–Bonferroni method.

*Post hoc* power analysis (Green and MacLeod, 2016; Brysbaert and Stevens, 2018) was carried out using simr::powerCurve() function to evaluate whether the sample size, i.e., the number of participants *N* = 10 was enough to detect an interaction effect between confidence and RT on aha feelings.

## Results

Of the total 650 trials, “Recognized” responses of object naming were observed in 619 trials (mean ± SD = 95.2 ± 4.7%), while “Don’t know” responses were recorded in the remaining 31 trials (4.8 ± 4.7%). Among “Recognized” responses, there were 580 trials (89.2 ± 5.8%) with correct answers and 39 trials (6.0 ± 4.7%) with wrong answers.

When examining the effect of the presentation change speed (Fast = 10 %/s vs. Slow = 5 %/s) on the Aha rating, the mean (± SEM) Aha score was not significantly different [*t*(9) = 0.41, *p* = 0.69] between Fast (3.42 ± 0.29) and Slow (3.46 ± 0.31) trials. Therefore, in the following analysis, data from the Fast and Slow trials would be merged without considering the presentation speed difference. Since there was no need to distinguish speed differences, RTs could be measured by ML units. In other words, hereafter, RTs and MLs would be regarded as interchangeable.

Mean subjective ratings were compared between correct and incorrect responses by the Type III ANOVA with Kenward–Roger’s degree of freedom correction (Table 1). Aha scores were significantly higher [*F*(1, 8.35) = 12.04, *p* = 0.032] in correct trials (mean ± SEM = 3.52 ± 0.29) than in incorrect (2.22 ± 0.49) ones, suggesting that the accuracy hypothesis was correct. Likewise, Pleasure [3.28 ± 0.28 vs. 2.08 ± 0.40, *F*(1, 8.30) = 16.26, *p* = 0.014], Suddenness [3.54 ± 0.28 vs. 2.29 ± 0.45, *F*(1, 8.38) = 11.84, *p* = 0.033], and Confidence [5.05 ± 0.25 vs. 2.64 ± 0.37, *F*(1, 8.43) = 46.84, *p* = 0.0004] ratings were significantly higher for correct responses than for incorrect ones (all *p*s reported here were corrected for multiple comparisons). Note that participants did not get any feedback about the correctness of answers during the trials. Although there were no external cues to confirm the correctness of answers, the subjects might have been able to accurately judge the likelihood of correct answer through the subjective feeling of correctness, as a subset of feelings related to metacognition. To investigate this possibility, we assessed the relationship between confidence and performance by type-2 ROC analysis (Fleming et al., 2010; Fleming and Lau, 2014) which defines the precision of metacognition as the type-2 AUC. The average AUC = 0.90 [95% CI = (0.81, 0.98)] was significantly higher [*t*(9) = 10.47, *p* < 0.001] than the chance level (i.e., 0.5 for random judgment).

**Table 1:** Means and standard deviations of subjective ratings for accuracy.

	Correct	Incorrect	Difference	*P* value
Aha	3.52 (0.93)	2.22 (1.55)	1.44 (1.19)	0.016^∗^
Suddenness	3.54 (0.89)	2.29 (1.41)	1.38 (1.15)	0.016^∗^
Pleasure	3.28 (0.90)	2.08 (1.27)	1.33 (0.95)	0.011^∗^
Confidence	5.05 (0.78)	2.64 (1.18)	2.33 (1.05)	0.0004^∗∗∗^

*Difference = Correct-Incorrect. Values inside the brackets represent SDs. ^∗^*p* < 0.05, ^∗∗∗^*p* < 0.001 two-tailed test with correction for multiple comparisons.*

In order to examine the relationship between performance and insight from another angle, we classified the solved trials into “insight” and “non-insight” to facilitate comparison with the previous research (Jung-Beeman et al., 2004; Danek et al., 2014a,b) in which dichotomous categorization were used (see discussion for the details of which scales or categorization should be used). Here, for simplicity, insight and non-insight were defined by Aha ratings of 4–6 and 1–3, respectively. The accuracy hypothesis and the confidence hypothesis were verified. We compared the mean accuracy of insight solutions (mean ± SEM = 98.2 ± 1.27%) to the mean accuracy of non-insight solutions (89.8 ± 2.69%). A significant difference [*t*(9) = 2.81, *p* = 0.02] was found, suggesting that participants were more accurate in insight solutions than in non-insight solutions. We also found a significant difference [*t*(9) = 7.26, *p* < 0.001] between the mean Confidence rating of insight solutions (5.28 ± 0.20) and that of non-insight solutions (4.44 ± 0.27), indicating that participants had higher confidence in insight solutions than in non-insight ones.

In what follows, further analysis would deal with only the correct responses, as the number of incorrect responses was found to be statistically insufficient. In addition, further analysis would be performed by treating Aha ratings as continuous (ordered multilevel) values instead of dividing into insight/non-insight.

We carried out correlation analysis in an exploratory manner to elucidate interrelationship between subjective ratings (i.e., “Aha,” “Suddenness,” “Pleasure,” and “Confidence” scores) and an objective measure (i.e., MLs). Aha, Suddenness, and Pleasure were positively and strongly associated with each other (all *r*_s_s > 0.7, multiple comparison adjusted *p*s < 0.001). Confidence was not correlated with Aha (*r*_s_ = 0.20, *p* = 0.35), but correlated with Suddenness (*r*_s_ = 0.37, *p* = 0.02) and Pleasure (*r*_s_ = 0.34, *p* = 0.03). MLs had a positive association with Aha (*r*_s_ = 0.33, *p* = 0.03) and a negative impact on Confidence (*r*_s_ = -0.52, *p* < 0.001). Detailed results including effect sizes (i.e., magnitude of correlations) and its 95% CIs of the correlation analysis are summarized in Table 2.

**Table 2:** Spearman correlation matrix for subjective and objective measures.

	Suddenness	Pleasure	Confidence	Morphing level
Aha	0.78^∗∗∗^	0.79^∗∗∗^	0.20	0.33^∗^
	[0.60, 0.89]	[0.61, 0.89]	[–0.11, 0.47]	[0.03, 0.58]
Suddenness		0.72^∗∗∗^	0.37^∗^	0.10
		[0.50, 0.85]	[0.05, 0.62]	[–0.18, 0.37]
Pleasure			0.34^∗^	0.06
			[0.03, 0.59]	[–0.19, 0.30]
Confidence				–0.52^∗∗∗^
				[–0.73, -0.23]

*^∗^*p* < 0.05, ^∗∗∗^*p* < 0.001 two-tailed test vs. 0. Values inside the brackets represent 95% confidence intervals with correction for multiple comparisons.*

**FIGURE 3:**
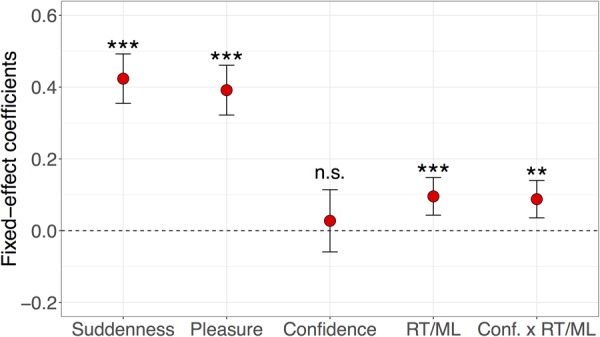
Factors contributing to the strength of aha feeling. Fixed-effect coefficients in hierarchical regression models that predict aha scores by utilizing linear mixed model (LMM) with participants as random effect. Error bars show the 95% confidence intervals. n.s. stands for not significant, ^∗∗^*p* < 0.01, ^∗∗∗^*p* < 0.001, two-tailed test vs. 0.

Although the rank correlation analysis revealed bivariate monotonic relationships between each pair of indices, it could not necessarily reflect relative influence because it did not consider multivariate entanglements, e.g., interaction effects. Therefore, in the next step as a complementary analysis, we utilized LMMs with the participant as a random effect and all other variables including their interaction terms as fixed effects in order to find out which factors determine the strength of the aha feeling. All variables were standardized into *z* scores for each participant level before applying the LMMs. As already mentioned, due to the compatibility between RTs and MLs, standardized MLs can be interpreted as standardized RTs (referred as RT/MLs). The coefficients of fixed effects are shown in Figure 3.

Suddenness and Pleasure were the two most influential factors determining the strength of Aha. In comparison, the influence of RT/ML and the interaction between Confidence and RT/ML were statistically significant but weaker than both Suddenness and Pleasure. Confidence alone was not significantly associated with the strength of Aha.

According to the power analysis, sample size *N* = 9 was sufficient to achieve power of > 0.8 in significance level = 0.05 to detect the interaction effect between confidence and RT/ML on the aha feelings. In the case of *N* = 10, which was actually the number we adopted in this study, the observed power was 90.2% [95% CI = (88.2, 91.8)].

**FIGURE 4:**
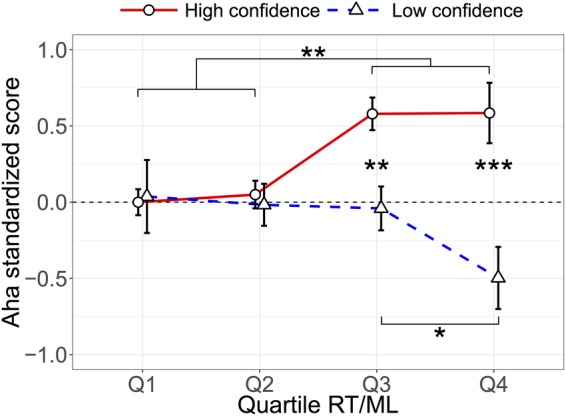
Aha *z*-score as functions of High/Low confidence and quartiled RT/ML. The mean *z*-scored aha rating as functions of subject-specific quartiles (Q1 = 0–25%ile, Q2 = 25–50%ile, Q3 = 50–75%ile, and Q4 = 75–100%ile) of response time/morphing level and split halves of confidence: High and Low confidence conditions corresponding to *z* Conf. > 0 and *z* Conf. < 0, respectively. Error bars show the standard error of the mean. ^∗^*p* < 0.05, ^∗∗^*p* < 0.01, ^∗∗∗^*p* < 0.001.

To further analyze the interaction effect on Aha between Confidence and RT/ML, standardized Confidence scores were divided into two categories: High confidence (*z* > 0) and Low confidence (*z* < 0), while standardized RT/ML scores were classified into quartiles. Both coarse-graining categorizations were performed by participant-wise manner. Consequently, grand mean Aha scores were calculated and plotted as functions of binarized Confidence and quartile RT/ML (Figure 4). We repeated the LMM analysis, focusing on only quartiled RT/ML as a fixed-effect factor, to High confidence and Low confidence conditions separately. In the High confidence condition, the strength of the aha experience was positively correlated with RT/ML (fixed-effect coefficient ± SE = 0.21 ± 0.06, *t*(8.79) = 3.35, *p* = 0.009). In contrast, that was not the case in the Low confidence condition (-0.15 ± 0.11, *t*(8.22) = -1.36, *p* = 0.21). In the first LMM, *z*-scored confidence was treated as a continuous (or at least ordered many-valued) variable. In the second LMM, High vs. Low confidence dichotomizing was applied just for simplicity to understand the interaction effect between confidence and RT/ML on aha feelings found in the first LMM. Due to the ceiling effect, dividing into “High” and “Low” confidence may therefore be interpreted as “ceiling high confidence” and “varied low confidence.” Note that categorization of RT/MLs using quartiles did not affect the results, because similar results were obtained if we used standardized RT/MLs as continuous variables as in Figure 3, instead of quartile RT/MLs. When comparing between High and Low confidence conditions, there were significant differences both in the 3rd and 4th quartiles (*p* = 0.002 and *p* < 0.001, respectively). Among the quartiles in the High confidence condition, the third and fourth were higher than the first and second (all *p*s < 0.01). Among the quartiles in the Low confidence condition, the fourth quartile was lower than the 3rd quartile (*p* = 0.03), and tended to be lower than the 2nd quartile (*p* = 0.056).

## Discussion

In the present study, we found that solving a hidden figure problem may evoke intense aha feelings along with feelings of suddenness and pleasure at the time of correct answer. In addition, the interaction between the time to solve and confidence is important to engender the aha experience. Strong sense of aha is likely to occur with a longer RT and a higher confidence in the absence of any feedback, suggesting a precise metacognition about one-shot learned knowledge.

Our finding that correct solutions are more likely to induce stronger aha feelings than incorrect responses is consistent with previous works showing that insight solutions, available in an all-or-none fashion, are correct more often (Salvi et al., 2016) than non-insight or analytic solutions, derived by conscious and incremental steps (Webb et al., 2016). In our analysis, high aha ratings and high suddenness scores directly reflect the all-or-nothing nature of insight solutions. The accuracy hypothesis and the confidence hypothesis were also supported by the results of grouping into insight and non-insight solutions.

To further consider potential effects of error types and rates on our interpretation of the results related to the accuracy hypothesis, we next conducted comparisons with the previous studies. According to Salvi et al. (2016), who investigated the accuracy hypothesis in various insight problems, only 6.3 and 2.4% of responded answers were incorrect (i.e., errors of commission) in the compound remote associates (CRA) and anagram problems, respectively. In our case, 6.3% of “Recognized” responses in hidden figures utilizing the GCP were incorrect, the commission error rate being comparable to the cases of CRA and anagram problems (Salvi et al., 2016). On the other hand, while timeouts (i.e., errors of omission) were observed in 52.3% of the CRAs and 27.0% of the anagrams in the previous studies, the omission error rate of hidden figures in the current GCP setting was only 4.8%, which was fewer than those of other insight problems. Our result was not inconsistent with the accuracy hypothesis. However, it remains unclear whether accuracy hypothesis applied to hidden figures will be still valid in the case of higher incidence of omission errors than the current level. Thus, alternative approaches to induce more errors of omission in the GCP to better test the accuracy hypothesis will be promising future directions.

High suddenness scores accompanying high aha ratings suggest that the subjects were not fully aware of the ongoing cognitive process of proper problem/solution representations toward problem solving until the very moment of an aha (Sandkühler and Bhattacharya, 2008). In this respect, insight can be characterized by lack of metacognition (Metcalfe and Wiebe, 1987) about the progress of processing before the sudden realization of solution.

It is also possible to consider the suddenness score as a subjective measure of cognitive processing fluency. The fluency theory of insight (Topolinski and Reber, 2010) predicts that high processing fluency leads to high degree of aha experience with strong positive emotions. We found strong positive relationships between suddenness, pleasure and aha feelings, consistent with the theoretical predictions of the fluency theory.

According to the fluency theory, high fluency is likely to induce high confidence. In the analysis within our experimental settings, a significant correlation between confidence and aha scores was not found. The lack of correlation might be due to the ceiling effect, masking the predicted interrelation. The ceiling effect is evident from the observation that about a half (48.5%) of all the confidence ratings was given the maximum rating of six.

The ceiling effect of confidence might be linked to higher accuracy and longer RT in the GCP paradigm, as the subjects could have adopted a “waiting strategy,” by continuing to watch a hidden figure movie until the stimulus uncertainty decreased enough and confidence became high enough to answer. The mean type II AUC (= 0.90) as an index of metacognitive accuracy (Fleming et al., 2010; Fleming and Lau, 2014) was well above the chance level (= 0.5) and close to the perfect score (= 1.0), suggesting that high “subjective” confidence was actually accompanied by high “objective” performance. Subjects had metacognition giving a significant prediction of the correctness of the answer in the absence of any feedback.

Another possible cause of the ceiling effect might be related to the usage of Likert scales in subjective ratings. There are several manners to measure self-report assessment of aha experience and related subjective aspects. The simplest way is to use dichotomous categorization of two-alternative forced choice, i.e., yes or no (Jung-Beeman et al., 2004). The way of presumably the most detailed way to discriminate nuanced difference is to use continuous, visual analogue scale (VAS) or detailed step division like scales from 0 to 100 (Webb et al., 2016; Danek and Wiley, 2017). There exist also studies which adopted combination of alternative measures: Danek et al. (2014a,b) used continuous measurement scales of insight-affective components (e.g., pleasure, confidence, etc.), as well as binary aha (yes or no), and outlined benefits of a more sensitive scale. Webb et al. (2016) advocated that continuous scales might be better than dichotomising categorization because in some cases in-between strength of insight/aha responses were indeed observed. Based on their suggestion, we prevented using dichotomous binary judgment paradigms. As a third option, methods with intermediate level measurement resolution between the binary judgments and the continuous scaling are the Likert scales, for example, 5-point (Bowden and Jung-Beeman, 2003, 2007) and 6-point scale (Tik et al., 2018). There continues a series of debates as to which of continuous VAS and discrete Likert scale is a better measurement method; Both of them have some advantages and also some disadvantages (for review, see Hasson and Arnetz, 2005). Usage of the Likert scales could limit the scale sensitivities to access ratings of each component of aha experience. We adopted, however, 6-point Likert scales as methods similar to Tik et al. (2018) rather than VAS to reduce task demand for respondents to move slide bars needed in case of the VAS. Recently, Simms et al. (2019) pointed out that “no psychometric advantages were revealed for any response scales beyond 6 options, including visual analogs” (VAS). Our choice of 6-point Likert scales was also consistent with their recommendation.

In our experimental settings, all stimuli were binarised and greyscale images of known objects. Comparison with control images with no object, e.g., stimuli with just pure noise, is one of interesting further research directions. The control condition would enable a “type-1” ROC analysis, which we could not conduct here, quantifying discriminative information of signal from noise. Even in such conditions with absence of any hidden objects, people tend to find something meaningful and try to make a partial or imperfect interpretation of objects that do not actually exist in the images (Whitson and Galinsky, 2008; Liu et al., 2014).

We assumed that binarised Mooney images were very difficult to interpret and original grayscale images were easy to recognize. If these assumptions were appropriate, stimulus change by constant speed between these Mooney and its grayscale counterparts would guarantee that RT or ML to recognize as a convenient measure of difficulty. However, median RT or ML as an index of difficulty diverged across hidden morphing movies. In general, there were various stimulus heterogeneity or stimulus dependency of hidden figures in gradual change paradigm (Ishikawa and Mogi, 2011) and it would be another source of difficulty. Further research is required to estimate and control the effects of stimulus dependency on the task difficulty.

In trials with short RTs (up to the median RT by subject), the mean standardized aha score was close to zero, whether confidence was high or not. Interpreting RTs as index of task difficulty, this result is consistent with Hebb’s (1949) theory, predicting that too easy problems cannot induce insight. The aha score did not become the lowest in trials with the shortest RT, however. The mean standardized aha level was not low enough but near zero, i.e., near the average score. One of the reasons behind this phenomenon might have been the tendency of the problem solver to be inclined to judge solution type impulsively as an insight when the RT was too short (Cranford and Moss, 2012; Salvi et al., 2016).

On the other hand, in trials with RTs longer than the median by subject, the average aha *z*-score was higher than zero for high confidence while the score was close to or lower than zero in for low confidence. This result is in line with the fluency theory of aha (Topolinski and Reber, 2010) which advocates that unexpected fluency gives high confidence and evokes positive emotions, i.e., an aha experience.

In our analysis, the strength of the aha feeling was not determined by RT/ML or confidence alone, but by both RT/ML and confidence. An interaction between RT/ML and confidence is an important determinant of aha feeling. Within the context of “confidence hypothesis,” it was found that the mean confidence was higher in insight (high aha rating) than non-insight (low aha rating) sessions. In GCP, because confidence and RT/ML was correlated, the relationship between aha and confidence might be also mediated by RT/ML. Thus, not the main effect of confidence, but the interaction effect between confidence and RT/ML on strength of aha was significant. In general, confidence levels are negatively correlated with RT/MLs (Ishikawa and Mogi, 2011), and the current result (Table 1) replicates the relationship; A negative relationship between confidence and RT/ML was found in the case of “by-item” analysis, or stimulus based averaging. It implies that participants tended to have, on average, lower confidence in difficult stimuli with longer/higher mean RT/MLs. In such difficult stimuli, the subjects could adopt a “waiting strategy” or viewing longer to get more information to recognize objects. Despite this general tendency, subjects can get high confidence with sudden realization even in trials with longer/higher RT/MLs. When such a special combination condition of RT/ML and confidence is met, the most intense aha feeling appears to occur.

Within the High vs. Low confidence group analyses, there are several possible interpretations. In the High confidence condition, positive relationship between aha and RT/ML indicates that (i) even in difficult stimuli requiring viewers to accumulate much more information (i.e., greater ML), participants are more likely to report a stronger aha experience if they finally get confident enough and/or (ii) people have high confidence and strong aha experience when the stimulus frames contain not enough information so that the viewer needs longer time (i.e., longer RT) to clearly recognize the image by information compilation, e.g., top-down knowledge. In the Low confidence condition, negative relationship between aha and RT/ML indicates that (iii) when it turns out that there is only insufficient information to be confident after viewing longer, people tends to have weaker aha experience or (iv) when people can recognize the image but not have confident about it, in general, they are less likely to report an aha experience. Note that the average aha *z*-scores were always equal to (i.e., not significantly different from) or lower than 0 in the Low confidence condition.

The results in this experiment are consistent with the incubation and restructuring theories of insight (Sandkühler and Bhattacharya, 2008), which advocate that waiting, struggling or stacked states called incubation period, mental fixation or impasse are needed before understanding problem deeply and reaching a solution to get out of the box. Long RT implies that such an incubation period, mental fixation or impasse exists before restructuring and recognition. In addition, several other factors, e.g., adapting a variety of solving strategies, may be involved in an extended RT.

As alternatives to solutions with insightful aha by intuitive and unconscious processing modes, several other solving strategies, such as trial and error, conscious, analytical, and deliberate thinking mode (Kounios and Beeman, 2009; Salvi et al., 2016) have been proposed. When faced with difficulty and disfluency, e.g., as a consequence of prolonged RT, subjects tend to switch dominant strategy from seeking insight to non-insight, e.g., analytical thinking mode (Alter et al., 2013; Thompson et al., 2013). This is consistent with Hebb’s (1949) theory, predicting that too difficult tasks do not always induce insight.

In the current GCP paradigm settings, movies of hidden figures are changing from degraded images (ML = 0%) to the blurred original images (ML = 100%) at a regular speed. In this setting, it is impossible to determine whether ML or RT is the more crucial metric. Interpreting RT as a measure of difficulty in our experimental settings has its limits, and should be applied with some caution. Further scrutiny is needed to clarify and dissociate effects of ML, ML change speed, and RT on aha feelings. Utilizing more flexible movie replay speed, for example, is one of several directions for future research. It would be interesting to investigate whether there exist optimal speeds or speed manipulation (e.g., acceleration) methods to induce an insight in GCP.

In summary, we successfully induced aha experiences accompanied by emotions such as surprise and delight in the perception of hidden figure in a laboratory setting by utilizing the GCP paradigm. It allowed us to identify the pivotal features in determining the strength of the aha. Specifically, the analysis suggests confidence and RT as metacognitive and temporal aspects contributing to the aha, respectively, with interaction between them. In conclusion, our findings provide metacognitive and temporal conditions for aha experiences, characterizing features distinct from those involved in non-aha cognitive processes.

## Author Contributions

TI and KM designed the experiments. TI and MT conducted the study and analyzed the data. TI, MT, and KM wrote and revised the manuscript and were critically involved in the interpretation of the results. All the authors listed have made a substantial, direct and intellectual contribution to the work, and approved it for publication.

## Conflict of Interest Statement

The authors declare that the research was conducted in the absence of any commercial or financial relationships that could be construed as a potential conflict of interest.
